# Biomaterials as a Vital Frontier for Stem Cell-Based Tissue Regeneration

**DOI:** 10.3389/fcell.2022.713934

**Published:** 2022-03-24

**Authors:** Ahmed Nugud, Latifa Alghfeli, Moustafa Elmasry, Ibrahim El-Serafi, Ahmed T. El-Serafi

**Affiliations:** ^1^ Pediatric Department, Aljalila Children Hospital, Dubai, United Arab Emirates; ^2^ Sharjah Institute for Medical Research, University of Sharjah, Sharjah, United Arab Emirates; ^3^ Department of Biomedical and Clinical Sciences (BKV), Linköping University, Linköping, Sweden; ^4^ Department of Hand Surgery and Plastic Surgery and Burns, Linköping University Hospital, Linköping, Sweden; ^5^ Basic Medical Sciences Department, College of Medicine, Ajman University, Ajman, United Arab Emirates

**Keywords:** stem cells, biomaterials, tissue regeneration, topography, differentiation, bioengineering

## Abstract

Biomaterials and tissue regeneration represent two fields of intense research and rapid advancement. Their combination allowed the utilization of the different characteristics of biomaterials to enhance the expansion of stem cells or their differentiation into various lineages. Furthermore, the use of biomaterials in tissue regeneration would help in the creation of larger tissue constructs that can allow for significant clinical application. Several studies investigated the role of one or more biomaterial on stem cell characteristics or their differentiation potential into a certain target. In order to achieve real advancement in the field of stem cell-based tissue regeneration, a careful analysis of the currently published information is critically needed. This review describes the fundamental description of biomaterials as well as their classification according to their source, bioactivity and different biological effects. The effect of different biomaterials on stem cell expansion and differentiation into the primarily studied lineages was further discussed. In conclusion, biomaterials should be considered as an essential component of stem cell differentiation strategies. An intense investigation is still required. Establishing a consortium of stem cell biologists and biomaterial developers would help in a systematic development of this field.

## 1 Introduction

Tissue engineering is an emerging field in medical care that links medicine to biology, engineering, physics and chemistry. The shortage of organ transplants as well as the limitations of artificial implants has enforced the research in this particular stream of regenerative medicine. The main goal of tissue engineering is the development of biological substitutes or functional constructs in order to restore or correct a defect in tissues or organs. Over the last few decades, human stem cells have become an attractive base for tissue engineering, with several case studies and proof of concept reports (Amini et al., 2012). Using stem cells in tissue engineering does not only open the potential up to produce specific tissues according to the patient’s need, but also reduces the risk of immune rejection. However, the full differentiation of stem cell populations into the desired target, is still an unmet challenge (El-Serafi, 2012). *In-vivo*, stem cells exist in an active and complex microenvironment; a fundamental factor of which is the extracellular matrix (ECM). The latter supports the cells and delivers physical and chemical clues that direct certain signaling pathways and affect the cell differentiation (Abagnale et al., 2015). Several biomaterials were designed to mimic the natural ECM effect *in-vitro* (Hellmund and Koksch, 2019). Furthermore, applying the biomaterials in 3D environment, can help in creating a human-based model that can reduce the use of animal in research (Nugud and El-Serafi, 2018).

**FIGURE 1:**
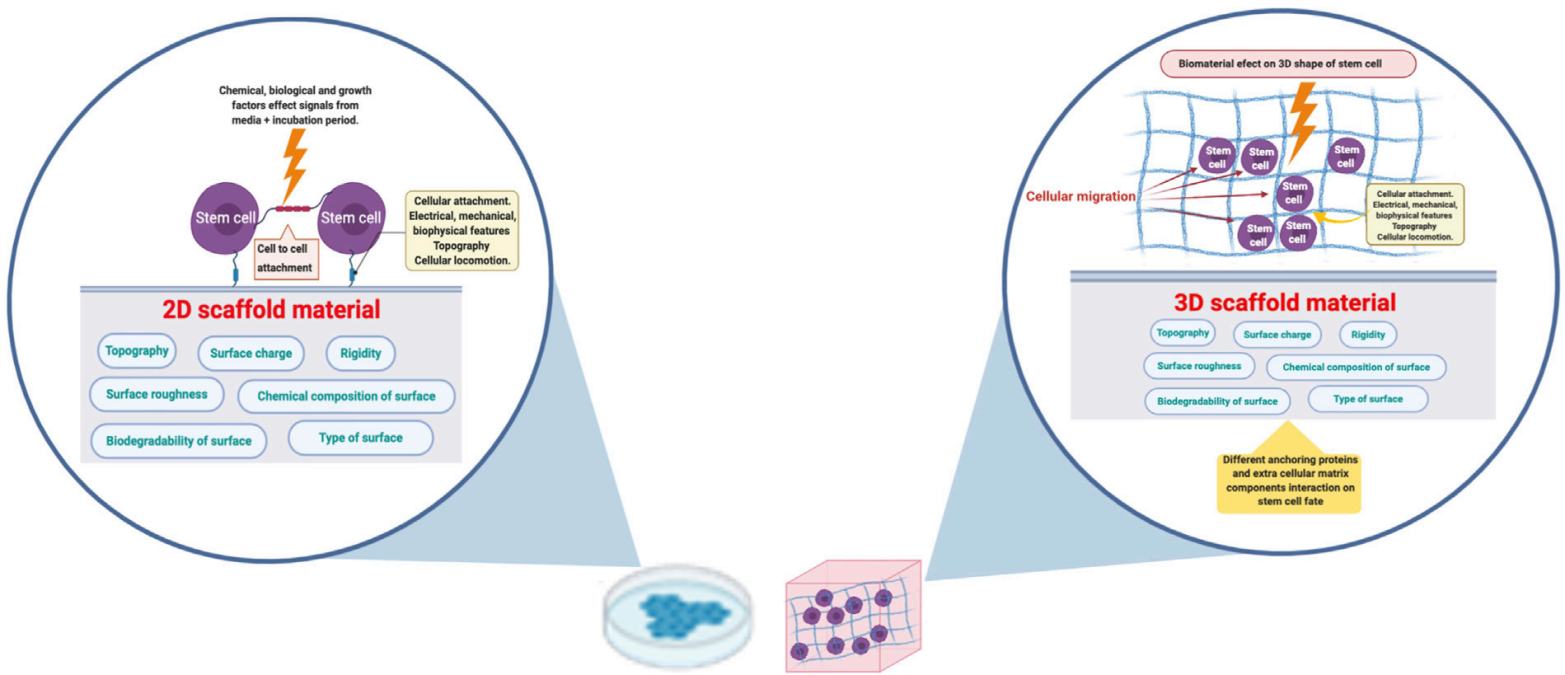
Applying a biomaterial in 3D configuration would enhance the cell-cell and cell-material interaction. The biomaterial surface charge, roughness, topography and chemical composition will affect the cells on the attachment boundary in case of 2D culture. Cells in 3D culture will receive the corresponding signals from the enclosing surroundings. The figure was created in BioRender.com.

In 2019, Williams define a biomaterial as “a material designed to take a form that can direct, through interactions with living systems, the course of any therapeutic or diagnostic procedure” (Williams, 2019). These materials need to be biocompatible in order to be applied *in-vivo*. Biocompatibility was defined earlier by Williams in 2008, as “the ability of a biomaterial to perform its desired function with respect to a medical therapy, without eliciting any undesirable local or systemic effects in the recipient or beneficiary of that therapy, but generating the most appropriate beneficial cellular or tissue response” (Williams, 2008). Biomaterials should be - at least - biotolerable; which is defined as the “ability of a material to reside in the body for long periods of time with only low degrees of inflammatory reaction” (Ratner, 2011). Ideally, biomaterials should be non-toxic, non-immunogenic, non-thrombogenic, non-carcinogenic and non-irritant. The early evaluation of biomaterials was restricted to the inert biocompatibility with the surrounding tissues or cells, although physical characteristics such as ultimate tensile strength, suture retention strength and stress–strain characteristics should also be considered. Metals were introduced to medical practice in bone fracture fixation around 1895. However, the use of metal in the field of biomaterials started to develop steadily when stainless steel was introduced for medical applications in early 1920s (Walker, 1912). Currently, there is a wide use of biomaterials in clinical practice, including hip prosthesis, steel rods for internal fixation, prosthetic heart valves, vascular stents, eye lens, contrast materials for magnetic resonance imaging as well as in dental materials and implants. The classical use of biomaterials in clinical practice are illustrated in Figure 1. Moreover, biocompatible polymers have also received special attention in the fields of biosensors as well as drug carrying and release (Bamford and Al-Lamee, 1992). Thus, it was not surprising for biomaterials to be involved in the tissue engineering technology (Williams, 2019). The ideal biomaterial should allow for functional replacement of tissue rather than providing simple physical support. Mostly are based on synthetic polymers, which should offer a combination of properties including good tensile strength and resemblance of the natural materials in the mechanical properties. Table 1 summarizes the most common application of biomaterials in clinical practice.

**TABLE 1:** Examples of biomaterials used to promote stem cell expansion and differentiation.

Lineage	Biomaterial	Notes	Reference
Cardiac	Native like hydrogels	MSC differentiation to cardiac lineage with MYH6 and CTnl expression	Lutolf et al. (2009)
	Hydrogel that stiffens over 300 h	Increase in cardiac differentiation of MSC by 60% and 3 fold increase in mature cardiac cell markers	Marin et al. (2020)
	Multilayer scaffold with 91.2% porosity	Neo-connective tissue and neo-vascularization with increase in angiogenic cytokines	Mahoney and Saltzman, (2001)
	Non-woven PGA with PCL\LLA polymer	IPSC differentiation to vascular smooth muscle cells with VEGF, PECAM and E-cadherin expression	Maturavongsadit et al. (2016)
	PGA mesh with silicon in pulsatile bioreactor	IPSC differentiation to vascular lineage with early smooth muscle markers expression and ECM deposition (Collagen I and II, fibronectin) Physical features similar to veins	Nour et al. (2019)
	3D Macro-porous nano fibrous PLLA scaffold with retinoid acid	Increase expression of α-SMA, MyoCD, SM22a, SMMHC	Monaghan et al. (2016)
Osteogenic	Ti6Al4V/Fibrin composite loaded with vascular endothelial growth factor	Significantly enhance osteogenesis	Nugud and El-Serafi, (2018)
	Stiff natural like hydrogels	Per-osteogenic markers permanently activated in MSC	Pennings et al. (2018)
	3D hydrogel with rapid relaxation rate	MSC differentiation to osteogenic lineage	Park et al. (2002)
	RGD modified alginate hydrogel	Four fold increase in osteoblast differentiation and proliferation, increased expression of osteocalcin	Pijnappels et al. (2008)
Chondrogenic	Polymer of PLGA, Chondroitin, hyaluronate	High potential of MSC chondrogenic differentiation	Samadian et al. (2020)
	Thermosensitive hydrogel copolymer of Chitosan, WSC-G-PNIPAAM	MSC chondrogenic differentiation forming tissue similar to articular cartilage	Sanz-Herrera and Reina-Romo, (2011)
Neuronal	PLGA with encapsulated nerve growth factor	Increase in choline aceyltransferase	Scopelliti et al. (2010)
	Three topographies of Chitosan	Porous and film topography promoted neuronal progenitors differentiation of MSC, high proliferation rate in 2D environment	Serrano-Aroca et al. (2018)
	Double layered Scaffold	Enhanced recovery of lower limb function after himisection of the spinal cord in animal model	Shahin et al. (2020)
	Collagen coated with polysaccharide with varying stiffness (soft and stiff)	MSC neuronal differentiation in soft material and osteogenic differentiation in rough material	Brown and Anseth, (2017)
	Hyaluronic acid hydrogel	Enhanced neuronal progenitor cells differentiation into astrocyte	Seale and Varghese, (2017)
	Electro-spun Poly (ε-aprolactone (PCL)	Enhanced neurite growth in neuronal stem cells	Seidlits et al. (2010)
Skin	Dextran based hydrogels	Increased rate of re-epithelization and nerve growth	Skardal et al. (2017)
	Heparin-hyaluronic acid hydrogel	Maintained a steady state release of growth factors in full burn murine model	Sun et al. (2012)
	Poly β aminoester scaffold	Enhanced MSC survival and via increasing angiogenesis	Pennings et al. (2018)
Stem cell expansion	Polyacrylamide hydrogels with varying side chains	MSC differentiation to osteogenic and myogenic lineage, Bone mineralization	Harrison et al. (2004)
	Polyhydroxyesteric\PLGA polymer	Alkali treatment of material surface enhanced the proliferation of mature ESC	Sundaram et al. (2014)
	3D biodegradable material pre-coated with fibronictin	Synergistic effect of mechanical stiffness to increase differentiation and integration of biomaterial into immuno-compromised animal model	Tay et al. (2010)
	Porous tantalum coated 3D scaffold	Supported hematopoietic stem cell growth in cytokines free media	Turner and Dalby, (2014)
	Tantalum coated porous biomaterial	Increase number of nucleated cells and colony forming units by 2.6 fold	Unal and West, (2020)
	Collagen I scaffold with Flt3 ligand and interlukin-3	Increase in number of colony forming units, upregulation of growth factor genes	Valamehr et al. (2008)

## 2 Types of Stem Cells

The term “stem cells” is very widely used and includes different types of cells with diverse differentiation abilities. Embryonic Stem cells (ESC) are pluripotent cells that can differentiate into all cell types that are derived from the three embryonic layers i.e. ectoderm, mesoderm and endoderm. This differentiation potential is limited in adult stem cells (ASC). These cells can be found in all tissues and are responsible for tissue homeostasis. The most famous subgroup of ASC is the mesenchymal stem cells (MSC), which are capable to efficiently differentiate into all cell types derived from the mesoderm. These cells can be isolated, with relative ease, from bone marrow, adipose tissue, and umbilical cord blood (Wang and Yeung, 2017). Furthermore, MSC can differentiate into cells derived from other embryonic lineages, based on the culture conditions, with variable degrees of success. For example, MSC was shown to differentiate into several types of cells including hepatocytes, beta cell of the pancreas, neurons and neuroectodermal cells (Cooke M. J. et al., 2010; Elsharkawi et al., 2019; Mansour et al., 2019; Khan et al., 2020). *In-vitro*, the proliferation of ASC, is limited by Hayflick’s limit, associated with senescence and decreasing ability to differentiate following few passages in culture. On the other hand, undifferentiated ESC preserves pluripotency and can give rise to teratomas. For this reason, any stem cell therapeutic approach should have efficient and safe differentiation towards the target cell type, which could be directed by the administration of the appropriate differentiation factors, as well as the use of specific biomaterial scaffolds and (Gadalla et al., 2018; Nugud et al., 2018). Nevertheless, these cells can enhance the natural capacity of tissue repair through their local secretions and release of exosomes with their micro-RNA (miRNA) content (Gentile and Garcovich, 2019).

Induced pluripotent stem cells (IPCs) are somatic cells transfected with potency genes that can provide the cells with the differentiation potential of ESC. Researchers considered IPCs as an important tool for understanding normal development, as well as disease onset, progression and new drug testing. The latter would involve creating disease specific IPCs and differentiate them into specific targets, in the presence of an appropriate scaffold, in order to create a 3D *in-vitro* model of diseased organ (Nugud et al., 2018).

## 3 The Interaction Between Stem Cells and Surrounding Environment

The main drive for *in-vitro* differentiation of stem cells is classically achieved via media supplementation with additives, such as growth and transcription factors. At the meantime, physical factors that exert regulatory effect on stem cells differentiation and proliferation remains to be clarified (Kshitiz et al., 2012). However, it is well-known from embryology that the morphogenetic cell movement and propagation result in the development of all body organs. Populations of progenitor cells undergo several processes whereby growing signals are gradually introduced, and they encounter a change in the cellular environment, resulting in cell division and expansion into tissues. In turn, some of these progenitors, along with stem cells, remain devoted to tissue turnover into organs. The control is based on cell-to-cell interactions and the molecular signals, adhesive signals and soluble matrix proteins present in the stem cell niche (Pennings et al., 2018). The interaction between ECM or biomaterials with stem cells revealed a potent effect on their differentiation, based on the adhesion and interaction between cells and underlying nanotopography. The latter is assembled at the scale of individual ECM molecules. The cells can also interact through contact guidance; a naturally occurring phenomena that regulate the cell orientation and movement based on the nature and shape of the surface. Similar effects can be obtained in biomaterials through designing grooves, pits, pores or other nanopatterns which can alter several cell characteristics, such as phenotype, survival, motility, proliferation, endocytic activity and gene regulation (Kshitiz et al., 2012; Driscoll et al., 2014). For example, Engler et al. (2006) showed that soft collagen coated with polysaccharides induced neuronal cell differentiation while the stiffer version induced osteogenic differentiation (Engler et al., 2006). The alterations of the cell behavior were explained by remodeling of the intranuclear factors that influence epigenetics (Kshitiz et al., 2012). Furthermore, several ECM proteins form large-scale structures (up to hundreds micrometers) can interact with multiple cells and organize a complex multicellular structure (Anderson et al., 2017).

## 4 Biomaterial Classification

Over the years, biomaterials have been classified according to different and overlapping approaches. Understanding these classifications could be crucial for the proper choice of a certain material for stem cell application.

### 4.1. Classification Based on Source

#### 4.1.1. Natural Source

Extracellular matrix components, such as fibronectin and collagen, represent natural resources for biomaterials that can be configured for medical and biological uses. Naturally, ECM provides a niche-like environment that accommodates and interacts with the surrounding cells. ECM components were shown to maintain cellular adhesion and promote cellular growth and differentiation of IPCs, *in vitro* (Dzhoyashvili et al., 2015). Natural biomaterials are known to be biodegradable and biocompatible, that give them an extra advantage. For example, decellularized extracellular matrix and lyophilized type I collagen were used for urinary bladder regeneration (Serrano-Aroca et al., 2018). Decellularization can be considered as a relatively safe procedure to produce a natural scaffold of the physiological nature of the original tissue (Gentile et al., 2020). Similarly, demineralized bone matrix allowed the integration into the bone defects and enhance endochondral ossification, thus, bone formation (Wang and Yeung, 2017).

#### 4.1.2. Synthetic Source

Synthetic biomaterials can be made of different forms of organic and inorganic materials, as well as different combinations of them. The chemical and physical properties can be changed according to the manufacturing process. In comparison to natural biomaterials, synthetic biomaterials have many advantages including; the possibility of standardization, the control of mechanical and chemical properties, degradation rate and byproducts, as well as the relative long shelf life, flexibility in shaping, possibility of bulk production and cost effectiveness (Prasadh and Wong, 2018). Regulation of degradation rate offers an important advantage that can be harnessed in tissue engineering to control cellular differentiation. On the other hand, cell attachment to synthetic materials can be less than the natural counterpart. Moreover, some synthetic biomaterials can induce host immune reaction. Examples of synthetic biomaterials include; 1) polylactic acid (PLA), 2) polyglycolic acid (PGA), 3) polycaprolactone (PCL), 4) poly lactide- co-glycolic acid (PLGA) (Cooke M. J. et al., 2010).

### 4.2. Classification Based on Bioactivity

#### 4.2.1. Bioinert Material

Bioinert materials interact with surrounding microenvironment, but not to the extent causing change in cellular structure or material at the light microscopic level. They support the surrounding cells and tissues during the repair process without chemical interaction. Clinically applied examples include polytetrafluoroethylene (PTFE), polymethylmethacrylate (PMMA), ceramics such as alumina and zirconia, and metals such as the titanium and cobalt-chrome-molybdenum alloys. As these materials have considerable strength and high wear resistance, they are commonly used as joint prosthetics including acetabular cups, femoral heads and dental protheses. Recently, bioinert hydrogels were characterized for three-dimensional tissue engineering (Kieswetter et al., 1996; Marin et al., 2020; Unal and West, 2020).

#### 4.2.2. Bio-Resorbable Material

Bioresorbable biomaterials are designed to provide temporary support, then get digested and absorbed through oxidation by free radicals, hydrolysis or through an enzymatic action, such as hydrolase, cholesterol esterase or phosphatases. Bioresorbable materials include sutures, stents and bone implants for the management of temporary clinical problems, such as narrowed arteries and fractured bones. These biomaterials are highly valuable in the field of regenerative medicine as stem cells should be able to differentiate and synthesis the natural extracellular matrix to replace the decaying biomaterial (Williams and Zhong, 1994; Ang et al., 2017; Cockerill et al., 2020).

#### 4.2.3. Bioactive Material

Bioactive materials interact directly with the surrounding cells and tissues on chemical basis. These materials are more likely to be based on composites that can be metabolized by the body, such as collagen-derived products, polylactic and polyglycolic acid polymers and processed bone graft (Brown and Anseth, 2017). Bioactive materials are either derived from natural resources, such as replamineform-treated hydroxyapatite compounds or synthesized as bioglass and synthetic calcium phosphates (Nour et al., 2019). The physical properties of these materials , including the surface composition, surface topography and surface charge would confer to their effects (Schwartz and Boyan, 1994). Bioactive glass has been very attractive for research and therapeutics in the field of orthopedics due to several characteristics, including osteoconductivty, as bioglass initiates precipitation of hydroxyapatite matrix on their surfaces and fibular nature. In addition, bioactive glass is used as a defect filler as it integrates with bony tissues and induces stem cell differentiation into osteoblasts *in-vivo* (Zhang et al., 2002). Furthermore, bio-active glass can stimulate osteoid formation by MSC directly and through enhancing neovascularization (Westhauser et al., 2019).

## 5 Effect of Biomaterials on Biological Organization

### 5.1. Protein Adsorption

Biomaterials surfaces are quickly coated with proteins in biological media. Adsorbed proteins could be related to the charge of the composite and the protein, material hydration at the interface and surface roughness of the biomaterials at the nanoscale. These factors contribute to a degree of selectivity for each material. As the protein surface integration happens, the molecular architecture of the surface change in correspondence of the orientation of ions, minerals, water and proteins. The expression of new cell adhesion molecules will result in change of the cell shape and behavior through the interaction with extracellular and intracellular signaling pathways (Scopelliti et al., 2010; Eliaz, 2019).

### 5.2. Cellular Adherence

Cell adhesion results mainly from the interaction between the biomaterial surface and the cell surface ligands. The composition of the material itself plays an important role in cellular attachment. Hanein et al., have shown in several studies the difference in the ability of renal cell adherence to calcium crystals, based on the three-dimensional orientation. The crystals were chemically equivalent, but structurally different, that was reflected on the crystallographic surfaces, as well as the cellular adhesion characteristics (Hanein et al., 1993; Hanein et al., 1994; Hanein et al., 1996). Cell adhesion can be enhanced by nanograting which resembles *in-vivo* extracellular matrix proteins. For example, mimicking collagen would allow the attachment and adherence of cells along the longitudinal axis. On the other hand, the reduction of cell attachment and constant filopodia formation can be guided by nanopits and nanoposts (Maturavongsadit et al., 2016). Skeletal stem cell depends on the adhesion formation and cellular spreading for functional differentiation, and that processes - at least partially - dependent on nanotopographical cues (Hart et al., 2007).

### 5.3. Cellular Motility

The concept of cell migration is based on cell movement pattern influenced by the biomaterial. The adherence between the cells and biomaterial as well as the elasticity of the biomaterial and the extracellular microenvironment, influenced by the protein adsorption, would promote cell motility. Historical results of Abercrombie et al., 1972, indicated that renal cells interacted with any surface through ruffled membranes, focal adhesions, adhesion plaques and focal contacts, which consequently affect the amoebic movement of the cells (Abercrombie et al., 1972). Cell motility occurs through the intra cellular scaffolding composed of microtubules, as well as intermediate filaments, and actin filaments (Sanz-Herrera and Reina-Romo, 2011). Early studies of cellular migratory behavior and the motility of chick heart fibroblasts, in terms of a velocity and direction of movement, was thought to be random (Abercrombie et al., 1971). Gail et al., in 1970, suggested that when mouse fibroblasts cultured on glass, they move neither in a perfectly uniform nor in a perfectly random way. Their studies demonstrated an intermediate tendency to persist in their direction of motion, which was supported latter by Abercrombie et al., in 1972 (Gail and Boone, 1970; Abercrombie et al., 1972). Contact guidance considered as a fundamental factor that regulates cell migration, which is naturally controlled by proteins of ECM. Moreover, contact guidance is crucial for organelle formation, including growth cone motility and axonal guidance (Discher et al., 2005; Al-Haque et al., 2012). For example, epithelial cells were reported to migrate from smoother to stiffer regions on biomaterials, in a phenomena known as durotaxis (Sanz-Herrera and Reina-Romo, 2011). Furthermore, cell migration would be affected by the surrounding environment *in-vivo.* The inflammatory mediators, produced by tissue macrophages, are normally present around the scaffold. These mediators attract endothelial cells and enhance their migration through the pores of the scaffold. From a regenerative perspective, this can help in the vascularization of the scaffold and enhance the construct sustainability. On the other hand, extracellular matrix secretion by the implanted cells can be negatively affected (Li et al., 2017; Saleh and Bryant, 2018). However, the interaction between cell-laden scaffolds and the surrounding environment is complicated and the ultimate effect can be induced by the scaffold or the enclosed tissue. Studying the migration of cells to and from the scaffold is both interesting and challenging, being affected by the method of labelling the cells, the timepoints of the study and the cell proliferation and migration.

### 5.4. Cell Proliferation

The interaction between the cell surface receptors and biomaterial surface can affect the cell proliferation. The chemistry-dependent interaction elicits a change in affinity specific integrin receptors, that affect gene expression via intracellular signaling pathways (Keselowsky et al., 2005; Lin et al., 2011).

## 6 Biomaterial–Stem Cell Interaction

The biomaterials are responsible for the formation of the stem cell microenvironment, where their physical, chemical and mechanical properties can influence cellular proliferation, integration and eventually differentiation. The use of biomaterials in stem cell differentiation has proved a great potential due to their specific properties that can guide differentiation and support the slowly forming tissue as well as the following biodegradability (Smith et al., 2010; Kumar et al., 2015). From a chemical perspective, the biomaterial important features consist of material composition, special ligands concentrations and possible biodegradability of the material. As for the physical properties, they involve the biomaterial mechanical properties, biomaterial topography, and morphology (Engler et al., 2006; Khetan et al., 2013).

### 6.1. Biomaterial Surface Topography Effect of Stem Cells

Biomaterials presented topographical landscapes like grooves, pits or pillars that can influence the proliferation, differentiation of different types of stem cells (Kshitiz et al., 2012; Driscoll et al., 2014). The focal contacts are specialized microstructures anchored to cytoskeletal microfilaments which play a role in shaping the cytoplasmic membrane. Components of the microstructure traverse the cytoplasmic membrane, allowing the cells to bind to the extracellular matrix via integrins. While the basal lamina in some types of tissues have unique nanofibrous characteristics, their substrate topography encounter functionality that interact with the cells. Similarly, synthetic topography was able to induce different effects on cells through affecting cell adhesion, alignment, morphology, proliferation, migration and even cytoskeletal organization (Schwartz et al., 1993). The influence of material topography on morphogenesis depends on the cell type as well as cytoskeletal organization, cell adhesion and the interaction between cells. Moreover, triggers from the extracellular environment can be transferred to the cell as a consequence of changes in the interaction of integrins, resulting in the stimulation or inhibition of intracellular signaling cascades with possible induction of new gene(s) and the synthesis of new protein(s) (Maturavongsadit et al., 2016). Focal adhesion kinase (FAK) would be activated and regulate ERK signaling pathway, which can affect both transcription and post-translational modifications that can lead to the differentiation of the stem cells in a machano-sensory approach (Biggs et al., 2008; Hamilton and Brickman, 2014; Cembran et al., 2020).

### 6.2. Biomaterial Surface Chemistry Effect on Stem Cells

Biomaterial surface chemistry can have a direct influence on stem cell differentiation. For example, side chains of polyacrylamide hydrogels would develop varying degrees of hydrophobicity without changing the mechanical properties of the gel. Such changes promoted -specifically - the differentiation of mesenchymal stem cells into osteogenic and myogenic lineages (Ayala et al., 2011). Similarly, high hydrophobic surfaces, such as octadecanethiol and polydimethylsiloxane, promoted differentiation of ESC toward the three germinal layers; endoderm, ectoderm, and mesoderm (Valamehr et al., 2008).

### 6.3. Host-Biomaterial Interaction

Implanted biomaterials can provoke an inflammatory response inside the body. This foreign body reaction could have deleterious effect on the chemical and biophysical properties of implanted biomaterials and alter its rate of biodegradability. This inflammatory response is considered as a major hurdle for the use of many biomaterials for *in-vivo* use (Sun et al., 2012). The description of host implant inflammatory response was introduced by Anderson et al., which included overlapping stages of injury, protein adsorption, acute inflammation, chronic inflammation, foreign body reaction, granulation tissue formation and encapsulation (Anderson, 1988). The acute phase of the reaction is dominated by neutrophils followed by macrophages within 48 h to in the latent phase of the reaction with the formation of foreign body giant cells (Henson, 1980). In fact, the host-macrophage response is an indispensable component of the constructive tissue remodeling process following the implantation of certain biologically derived scaffolds (Badylak et al., 2008; Valentin et al., 2009; Brown et al., 2012; Brown and Badylak, 2013). The type of adsorbed proteins onto the composite surface in addition to the surface chemistry of the material and the topographic features are all considered to be important and contributing factors in the severity of the host response (Anderson et al., 2008; Chen et al., 2010). Henceforth, the success of implanted biomaterials is dependent on evasion or control of inflammatory response either by implanting biocompatible materials or inhibiting the immune response (Ye et al., 2011).

The interaction between the host and biomaterial can affect the properties of the latter. For example, contact with body fluid can enhance the degradation rate which alter the material density as well as its volume and strength. This effect can be induced by the *in-situ* environment and the change in pH, the presence of certain enzymes and electrolytes as well as the mechanical forces around the biomaterial, such as the weight, muscle contraction, joint movement, and shear stresses (Wang et al., 2019).

### 6.4. Two Dimensional Versus Three Dimensional Cell Culture for Stem Cells

Monolayer or two dimensional (2D) cell culture is the most convenient way of cultivating cells *in vitro* and it is carried out on flat surfaces with cells exposed to soluble elements in the media. This culture system is a simple method to dissect the role of individual components on stem cells differentiation (Lutolf et al., 2009). On the other hand, three dimensional (3D) microenvironment could provide more insight into an *in-vivo* like cellular behavior, including the cell shape and interactions, as well as enhancement of gene expression (Nugud and El-Serafi, 2018). For example, 3D culture allows cells to better communicate and migrate that can promote cellular differentiation (Figure 1) (Baharvand et al., 2006). Stem cells in particular are extremely sensitive to any change in the microenvironment. We have previously shown the difference in gene expression for stem cell differentiation into adipogenic lineage between monolayer and in a scaffold-free 3D culture. The latter method has clarified the upregulation of the relevant gene expression between the differentiated and control cells, which was not obvious in monolayer culture (El-Serafi et al., 2019). Stem cells cultured in 3D constructs, can differentiate and self-organize into a multi-type cell construct (El-Serafi et al., 2011; Sozen et al., 2018). Moreover, the presence of suitable biomaterials could enhance the cell “self-assembly” into larger and transferable constructs (Jean et al., 2011). Similarly, stem cells cultured on electrospun-nanofibrillar polyamide 3D scaffold had superior proliferation and migration potential when compared to a 2D model using the same biomaterial (Nur et al., 2006). Thus, the ideal scaffolds design should be complex enough to mimic native tissue architecture as well as allow cellular attachment, migration, proliferation and differentiation, taking in consideration the material physicochemical properties (Kloxin et al., 2009; El-Serafi et al., 2017; Nugud et al., 2018). Such approach will greatly help in the understanding of complex cellular pathways.

## 7 Examples of Biomaterial Effect on Different Stem Cell Population

### 7.1. Cardiac and Vascular Differentiation

Native-like hydrogels, resembling *in-vivo* environment of the heart, were able to induce cardiac differentiation of MSC during a 2 weeks *in-vitro* culture. Around 80% of MSCs expressed cardiac myosin and troponin, which are important markers involved in cardiac cells contraction-relaxation process (Li et al., 2012). This method had a higher cardiac differentiation potential when compared to commonly used modalities such as co-culture with cardiac cells or using epigenetic modifiers such as 5-azacytidine (Pijnappels et al., 2008). In another model, human MSC were cultured as a multilayer and loaded on a porous acellular bovine scaffold and implanted in a rat myocardial infarction model. Native-like neo-connective tissue fibrils and neo-vascularization were developed and angiogenic cytokines (bFGF, vWF, and PDGF-B), cardiac markers (Nkx2.5 and MEF2D), and cardiac protective factors (IGF-1 and HGF) were expressed in the transplanted construct (Wei et al., 2008). The combination of stem cells and biomaterials would not only promote cardiac tissue repair and homeostasis, but also guide the differentiation of the 3 cell types in the heart wall; i.e., cardiomyocytes, smooth muscle, and endothelial cells. Different types of stem cells were involved in these studies, with particular success with cardiac progenitor cells, isolated from healthy cardiac tissue and identified with one of the cell surface markers c-Kit, Sca-1, CD31 and Flk-1. The biomaterials used are extracellular matrix protein-based, decellularized matrices and several polymer-based materials. These biomaterials can retain the intrinsic biotic activity to support cell adhesion, differentiation, and subsistence (Cutts et al., 2015). Furthermore, Young et al., aimed at mimicking cardiac development by creating a hydrogel that stiffens over a time period of 300 h after cell seeding. The authors reported an increase in functional cardiac muscle fibers by 60% and a three-fold increase in number of mature cardiac cell markers, indicating improvement of cardiomyocytes differentiation by the gradual stiffness of this biomaterial over time (Young and Engler, 2011).

MSCdifferentiation into cardiomyocytes could be influenced by biomaterial micro-topography. Coating PLGA material with ECM protein and human plasma derived fibronectin allows the formation of different topographies and forming spatially defined geometries. The latter promoted differentiation towards a cardiac muscle lineage, by induction of certain genes including; GATA4, MyoD1 which are early regulators of cardiomyogensis as well as β-MHC and cTnT genes (Tay et al., 2010). Hibino et al., in 2012, reported the differentiation of iPSCs to the vascular lineage on a flat non-woven porous PGA mesh and a co-polymer sealant solution of e-caprolactone and l -lactide scaffold. The differentiation was noted by expression of vascular and endothelial markers; VEGF, PECAM, and E-cadherin (Hibino et al., 2012). For blood vessel tissue engineering, murine stem cells were cultured on a macro-porous nanofibrous, polylactide scaffolds, in the presence of retinoid acid. The scaffold promoted stem cell differentiation into smooth muscle cells, by mimicking the architecture of vascular tissue, with expression of the early and late smooth muscle markers such as; α-SMA, MyoCD, smoothelin, SM22a, and SMMHC (Xie et al., 2011). In a pulsatile bioreactor, human iPSCs were seeded on PGA mesh over a silicone tube for 8 weeks. The cells expressed early stage smooth muscle markers including; α-SMA, SM22α and calponin, while the absence of the osteochondrogenic markers were absent. The extra cellular matrix was positive for the presence of collagen I, collagen II, and fibronectin. Interestingly, engineered vessel could withstand the pressure up to 700 mmHg, which is approximately half that of the normal veins pressure (Sundaram et al., 2014).

### 7.2. Osteogenic Differentiation

The progress in biomaterials development in relation to osteogenic differentiation is critical to the advancement of both dentistry and orthopedics. The use of proper biomaterial can help the healing process through the recruitment of MSCs and the stimulation of their differentiation (Logan and Brett, 2013; Lv et al., 2015). Materials used as a substitute or a fixative for bone should provide mechanical support and promote weight bearing as well as serving as a medium for osteo-regeneration. These biomaterials should be, 1) osteoconductive; i.e., able to support the attachment of osteoblasts and the promotion of bone formation on its surface, including neovasculogenesis, 2) osteoinductive; promote the expression of bone specific markers and osteogenic proteins in response to the biomaterial 3D configuration, chemical and physical composition, followed by mineralization and calcification of newly formed bony tissue; and 3) supports osteogenic differentiation of progenitor cells, osteoblasts, and bone marrow stromal cells into mature osteoblasts (Agrawal and Srivastava, 2020). Thus, an ideal bone biomaterial would be biocompatible, bioactive and biodegradable, such as bioceramics, polymers, and metals (Gao et al., 2017). *In-vitro*, the mechanical properties of a biomaterial can affect the osteogenic differentiation. For example, MSCs showed faster proliferation and osteogenic differentiation when cultured within 3D hydrogels with rapid relaxing rate; i.e. relaxing time of about 1 min (Chaudhuri et al., 2016). When MSC were cultured on stiff hydrogel before being transferred to soft hydrogel, the pre-osteogenic transcription factors (YAP, TAZ and Runx2) were permanently activated (Yang et al., 2014). Similarly, the physiochemical properties can affect the osteogenic differentiation. When the osteoblast cell line “MC3T3 -E1” were cultured on RGD-modified alginate hydrogel, both the proliferation rate and osteoblastic differentiation were enhanced, including a 4-fold increase in osteocalcin, a late stage osteoblastic differentiation marker (Lee et al., 2004). Micro and nano-topography, produced by electron beam lithography, colloidal lithography and polymer demixing techniques, were able to promote the differentiation of osteogenic of human MSCs. Nanograting can influence the polarization of MSCs by contact interaction and osteogenesis promotion. The symmetry and order of the nanopits enhanced the expression of bone specific ECM proteins, such as osteopontin and osteocalcin. When MSCs were cultured on completely random nanopits, no expression of both markers could be detected (Turner and Dalby, 2014; Aminuddin et al., 2016).

### 7.3. Chondrogenic Differentiation

Micro aggregation, along with progenitor cells’ condensation, are the main events that create a specialized microenvironment drives chondrogenesis during bud development. Thus, the biomaterials involved in chondrogenesis should contain components that help to establish this microenvironment for the cells and hence promote their microaggregation and chondrogenesis (Leijten et al., 2016). Many natural and synthetic biomaterials have been investigated their chondrogenic differentiation potential, including synthetic and natural silk, cellulose, marine sponge fiber skeleton, hyaluronan and hyaluronic acid, in addition to a hybrid polymer of synthetic and natural material. The combination of PLGA, gelatin, chondroitin and hyaluronate provided a greater potential in repairing full thickness cartilage defects in rabbits when compared to PLGA biomaterial alone (Fan et al., 2006). Injectable polymers loaded with cells represent an intense area of research with potential for clinical application as it can be easily introduced to the joint space. In a preclinical trial, a thermosensitive hydrogel was loaded with human MSCs and injected into the bladder submucosal layer of rabbits. As the gel underwent structural changes by the effect of the body temperature, the new confirmation enhanced MSC chondrogenic differentiation and formed a tissue that resembled articular cartilage with a mixture of hyaline and fibrous cartilage (Cho et al., 2004). On the other hand, the material stiffness has been shown in another system to affect the chondrogenic differentiation. When MSC were cultured on a rigid surface-charged methyl acrylate/methyl methacrylate, the osteogenic differentiation prevails. Culturing the same cells on the lower rigidity substrate enhances chondrogenesis (Yoon et al., 2018). Other biomaterials, such as fibrin glue, type 1 collagen gel, peptide hydrogels, and Matrigel, have been also studied for *in vitro* chondrogenesis with MSCs and resulted in a cartilage-like transplants (Monaghan et al., 2016).

### 7.4. Neuronal Differentiation

The role of biomaterials in peripheral nerve regeneration is crucial. In addition to enhancing the glial and tubular cell differentiation, the material physical conformation can guide the nerve growth direction. Several natural biomaterials have been tested for nerve tissue regeneration, including collagen, gelatin, chitin, elastin as well as hyaluronic acid. Synthetic biomaterials, such as poly-3-hydroxybutyrate, have been also used for neuronal repair, with the advantage of incorporating several neurotrophic factors during the manufacturing process (Mahoney and Saltzman, 2001; Seidlits et al., 2010; Wang et al., 2012; Samadian et al., 2020). Moreover, the neurogenic differentiation can be affected by the several physical and mechanical factors, based on the nature of the material and the surface topography. Additionally, stiffness of the biomaterial is a key influential factor for neurogenesis. MSC cultured on soft polyacrylamide hydrogel (∼0.5 kPa) were directed towards neurogenesis, while the stiffer substrate (∼40 kPa) would favour osteogenesis (Yoon et al., 2018). Wang et al. (2010) have studied the differentiation of neural stem cells (NSC) on chitosan on three different topologies; film, porous scaffold and multi-microtubule conduit. In the film topology and multi-microtubule conduit, cells were tightly adherent and elongated, exhibiting a star-like morphology with a network of inter-communicating processes that supported the differentiation to neuronal linage. On the other hand, porous scaffold did not drive NSC to differentiate. Gene expression study and immunostaining were performed for glial fibrillary acidic protein (GFAP; an astrocyte marker), b-tubulin III (an early neuronal marker), and O1 (an oligodendrocyte marker). The authors highlighted induction of GFAP, a mature astrocyte marker, in cells cultured on films, indicating a preference towards astrocytes differentiation. Cells seeded on porous scaffold and multi-microtubule conduit had an induction of b-tubulin III, suggesting differentiation toward neuronal lineage. However there were no significant differences in O1 gene expression, an oligodendrocyte marker, among the three experimental groups (Wang et al., 2010).

The combination of biomaterials allows further complexity of the constructs. For example, a double layered scaffold that is similar to white and gray matter of spinal cord, was seeded at the inner part with stem cells. The cells embedded in the biomaterial were transplanted into a hemisectiond spinal cord lesion in a rat model, which showed enhanced recovery of the lower limb neurological function in comparison to control subjects with cells or scaffold alone. The results were attributed to diminished glial scarring, reduction of tissue loss due to the injury, and the reestablishment of axonal connection. The same group reported later that an implanted glycolide scaffold seeded with stem cells was able to establish a two ways interaction between brain and viable areas in an ischemia-induced lesion model (Park et al., 2002; Teng et al., 2002).

### 7.5. Skin

The role of biomaterials in skin regeneration has been previously established in the clinic, as a temporary cover for extensive wounds, with improved re-epithelization, matrix formation and nerve growth. The list of examples includes dextran hydrogels, biodegradable polymers and bioactive glass microfibers (Martin et al., 2014; Shen et al., 2015; Zhou et al., 2016). Several biomaterials were acquired to the field of skin regeneration, using patient-derived keratinocytes or stem cells (El-Serafi et al., 2017). Skardal et al. (2017) designed a heparin-hyaluronic acid hydrogel, loaded with amniotic fluid-derived stem cells. This construct was introduced to a full thickness burn wound model and was associated with better production of extra-cellular matrix, increased rate of revascularization and eventually better rate of re-epithelialization. The authors relate these results to the ability of their hydrogel to sequester and release the paracrine secreted growth factors and cytokines, which prolonged their effect (Skardal et al., 2017). Various types of synthetic biomaterials are available as dermal substitute (DS) for clinical use. The main role of DS is to replace the lost skin until the patient is prepared for skin grafting, which fastens the wound healing and enhance the angiogenesis process (De Angelis et al., 2015; Gentile and Garcovich, 2021). Collagen-based DS showed efficiency also in skin ulcers of vasculogenic origin (De Angelis et al., 2019). Alternatively, animal derived, decellularized dermis can be used in severe cases, such as loss of a large surface area of skin (Gentile et al., 2013). In a different approach, autologous fat-derived scaffold can be considered as a clinical solution for wound and scar care with better skin quality and healing time. Fat tissue can be mechanically or enzymatically processed to provide adipogenic derived stem cells suspended in fatty tissue of the stromal vascular fraction, which contains other cell types such as endothelial cells, pericytes and immune cells (Gentile et al., 2017; Gentile et al., 2021). Similar approach was used in breast reconstruction following mastectomy instead of an implant with safe and effective clinical outcome (Gentile et al., 2019).

### 7.6. Biomaterial Assisted Expansion of Stem Cells

A major challenge with stem cell implementation for therapeutic approaches is to obtain enough yield of cells with a good quality, in an environment free from animal-derived products using reproducible technique. Multiple studies investigated the effect of biomaterials on stem cells, in terms of proliferation and preservation of their phenotype. For example, alkali treatment of PLA and PLGA on murine ESC increased the material hydrophilicity and the cell proliferation (Harrison et al., 2004). Furthermore, scaffold stiffness acted synergistically with fibronectin coating to enhance ESC differentiation compared to the less stiff matrigel scaffold which supported the growth of ESC (Levenberg et al., 2003). Similarly, vitronectin contained biomaterials provided a suitable matrix for the adherence and expansion of iPCS (Seale and Varghese, 2017). The same effect can be obtained for hematopoietic stem cells when cultured in a porous scaffold coated with tantalum that resembled the microarchitecture of bone marrow trabeculae without the need of any cytokines (Bagley et al., 1999; Ehring et al., 2003). The same type of stem cells showed enhanced colony formation and upregulation of growth factors-related genes when cultured on scaffold of collagen type 1 in the presence a cocktail of ligands (Oswald et al., 2006). Thus, each type of stem cell may require a special biomaterial to enhance its proliferation, without inducing differentiation.

## 8 Biomaterials and Complex Tissue Organization

Most of the body tissues consist of different types of cells that interact in a harmonized organization. Such interaction is not only responsible of the sustainability of the tissue, but also for the functionality. For example, bone and cartilage are present adjacent to each other in a well-defined configuration at the articular surface of the joints. This formation allows weight support as well as protection of the bone ends from erosion; thus a cartilage defect can be easily extended to the underlying bone. The 3D scaffolds, in this case, can be tailored to be a composite of two types of biomaterials which correspond to the two target tissues. Not only the material, but also the fabrication properties would differ according to the target tissue (Jia et al., 2018). Vascularization is another challenge for cell-biomaterial constructs for maintaining its viability *in-vivo* (Shahin et al., 2020). In classical scaffold-based 3D culture system, neovasculogenesis is dependent on the media supplement. Unfortunately, the osteogenic supplements in media do not support elongation of new vessels, while the angiogenic factors decrease the osteogenic differentiation ability (Schott et al., 2021). On the other hand, self-assembly bone construct can be associated with spontaneous chondrogenic matrix formation and the extension of capillary like structure. The biomaterial used in this case was glass, which served as a surface to allow folding of the monolayer in a 3D bony construct (Alghfeli et al., 2021).

Skin represents another example of complicated multilayer structure. Bioprinting and electrospinning provide a solution through the preparation of a multilayer scaffold that would support the attachment and survival of both keratinocytes and fibroblast to represent the epidermal and dermal components (Choi et al., 2021). The multilayer scaffold should take into consideration the physical properties of each biological layer. For example, stiffness gradient differs between the skin layers can be reflected on the amount of calcium ions at the corresponding level of the scaffold. Calcium is responsible for the activation of several intracellular signalling cascades and consequently can involve the determination of the cell fate (Chaudhari et al., 2016). In conclusion, combination of biomaterials or combination of different physicochemical properties of the same biomaterial may enhance the bioengineering of complex tissues.

## 9 Conclusion and Recommendations

The field of biomaterials has already proved its importance in the field of stem cell biology, as well as differentiation and potential medical applications. There are many technical challenges that yet to be solved, such as the adherence of seeded cells to the bio-scaffold and integration with host tissue, as well as cytotoxicity as a result of amplified immune response from immune system or inflammatory response. Accordingly, biomaterials should also provide a sort of isolation to the cells seeded in them. Establishing specific substrates for cell expansion or differentiation into various lineages will only not have a great biological benefit but also economical interest, which would result from avoiding the expenses of different peptides and growth factors. Another major challenge is the production of complex scaffolds that can co-culture multiple cell lines. There is vast number of studies that proved the biomaterial-stem cell interaction with or without fully explaining the underlying mechanisms. Protein adsorption, cell adhesion and sensing the underlying structure, signaling pathway(s) activation, gene response and differentiation are the generic processes. However, few studies compared the effect of various compositions, forms or nanostructure and topography of the same biomaterial on the proliferation versus the differentiation potential of stem cells into different lineages. A proper description of the biomaterials according to their chemical and physical properties in relation to their effect on stem cells will be a major addition to the regenerative medicine. Furthermore, a proper description of the role of these biomaterials as additive to the current differentiation protocols or as a replacement of one or of the differentiation components can have a great biological impact as well as medical and commercial applications. Thus, a consortium of stem cell biologists and biomaterial developers and producers is highly needed in order to investigate a matrix of available and in-development materials against various types of stem cell differentiation and proliferation. Such collaboration can enhance the progress of application of stem cell-based tissue regeneration in the clinic.
